# The inner side of yeast PCNA contributes to genome stability by mediating interactions with Rad18 and the replicative DNA polymerase δ

**DOI:** 10.1038/s41598-022-09208-7

**Published:** 2022-03-25

**Authors:** Robert Toth, Miklos Halmai, Zsuzsanna Gyorfy, Eva Balint, Ildiko Unk

**Affiliations:** The Institute of Genetics, Biological Research Centre, Szeged, Eotvos Loránd Research Network, Szeged, 6726 Hungary

**Keywords:** Genetics, Molecular biology

## Abstract

PCNA is a central orchestrator of cellular processes linked to DNA metabolism. It is a binding platform for a plethora of proteins and coordinates and regulates the activity of several pathways. The outer side of PCNA comprises most of the known interacting and regulatory surfaces, whereas the residues at the inner side constitute the sliding surface facing the DNA double helix. Here, by investigating the L154A mutation found at the inner side, we show that the inner surface mediates protein interactions essential for genome stability. It forms part of the binding site of Rad18, a key regulator of DNA damage tolerance, and is required for PCNA sumoylation which prevents unscheduled recombination during replication. In addition, the L154 residue is necessary for stable complex formation between PCNA and the replicative DNA polymerase δ. Hence, its absence increases the mutation burden of yeast cells due to faulty replication. In summary, the essential role of the L154 of PCNA in guarding and maintaining stable replication and promoting DNA damage tolerance reveals a new connection between these processes and assigns a new coordinating function to the central channel of PCNA.

## Introduction

Proliferating cell nuclear antigen (PCNA) is an evolutionarily highly conserved protein. It has a central role during DNA replication that is to provide a sliding platform to the replicative DNA polymerases epsilon (Polε) and delta (Polδ)^1–4^. The homotrimer ring-shaped PCNA molecule encircles DNA and tethers the connecting replicative DNA polymerase, preventing its dissociation from DNA^5,6^. For example, yeast Polδ synthesizes only a few nucleotides in the absence of PCNA before falling off, whereas it can synthesize more than 5 kilobase-long runs in a single binding event with PCNA^7,8^. In addition to replicative polymerases, other non-classical DNA polymerases taking part in translesion synthesis also bind PCNA, and their processivity and synthetic activity are enhanced by the binding, although to a much lesser extent compared with Polδ^9–12^. PCNA interacts with several members of the replisome as well, and it serves as a docking place for and coordinates the activities of proteins participating in different aspects of DNA metabolism like DNA repair, cell-cycle control, chromatin assembly^13^. PCNA can fulfill regulatory functions through its posttranslational modifications, as well. For example, the Rad52 dependent homologous recombination and the Rad6/Rad18-governed DNA damage tolerance (DDT) are controlled by the sumoylation and the ubiquitylation of PCNA, respectively^14,15^. During normal replication, the K164 of PCNA is modified by the Ubc9 SUMO conjugase and by the SUMO ligase Siz1. Sumoylated PCNA binds the Srs2 helicase that disassembles Rad51-formed filaments on single-stranded DNA at the replication fork, thereby preventing unscheduled recombination that could lead to genomic rearrangements^16–18^. DNA damage tolerance comes into play when replication stalls at a DNA lesion. The Rad6 ubiquitin conjugase together with the Rad18 ubiquitin ligase regulates at least three subpathways in response to ultraviolet light (UV)-induced replication blocking DNA lesions^19,20^. By monoubiquitylating PCNA on lysine 164 (K164), they activate two subpathways that depend on translesion synthesis (TLS) polymerases^21^. One of them is mainly error-free and is carried out by the *RAD30*-encoded DNA polymerase η, and the other is error-prone and is executed by Rev1 together with DNA polymerase ζ consisting of the Rev3 and Rev7 proteins^22–24^. The third sub-pathway is turned on by further polyubiquitylation of the same PCNA residue by the Mms2/Ubc13 ubiquitin conjugase and the Rad5 ubiquitin ligase, and it operates through template switching, during which the information of the newly synthesized sister chromatid is used for bypassing the lesion^25^.

Most PCNA interacting proteins bind the hydrophobic pocket formed by the interdomain connecting loop (IDCL) of PCNA through a conserved PCNA interacting protein (PIP) motif^26,27^. However, the C-terminus and the subunit interface also serve as binding sites to several of its partners^28–32^. Surprisingly, the inner side of the PCNA ring also mediates protein interaction, as it was shown to form part of the binding site for the PCNA-associated factor p15 using human proteins^33^. The surface of the PCNA ring facing the negatively charged DNA is composed of alpha-helices with a positive overall charge. It maintains a dynamic connection with DNA and ensures the sliding of PCNA on the double helix. Recently, it has been assigned regulatory functions, as well, since acetylation of lysine residues at the inner surface has been shown to promote sister chromatid mediated homologous recombination for DNA repair, and removal and degradation of chromatin-bound PCNA during nucleotide excision repair^34,35^.

In this study, we characterize a yeast PCNA mutant that carries a single amino acid change at the inner side of PCNA. We show that the mutation completely inactivates the Rad6/Rad18-dependent DTT by preventing DNA damage-induced PCNA ubiquitylation via hindering the binding of Rad18 to PCNA. Moreover, the mutation also abolishes PCNA sumoylation. In addition, it perturbs replication by altering the interaction between PCNA and Polδ, leading to frequent dissociation of the replicative DNA polymerase and thereby to misalignment of the nascent DNA end at mononucleotide repeats, resulting in genome instability.

## Results

### The *pol30-L154A* yeast strain is slow-growing and exhibits a DNA damage sensitive phenotype

**Figure 1:**
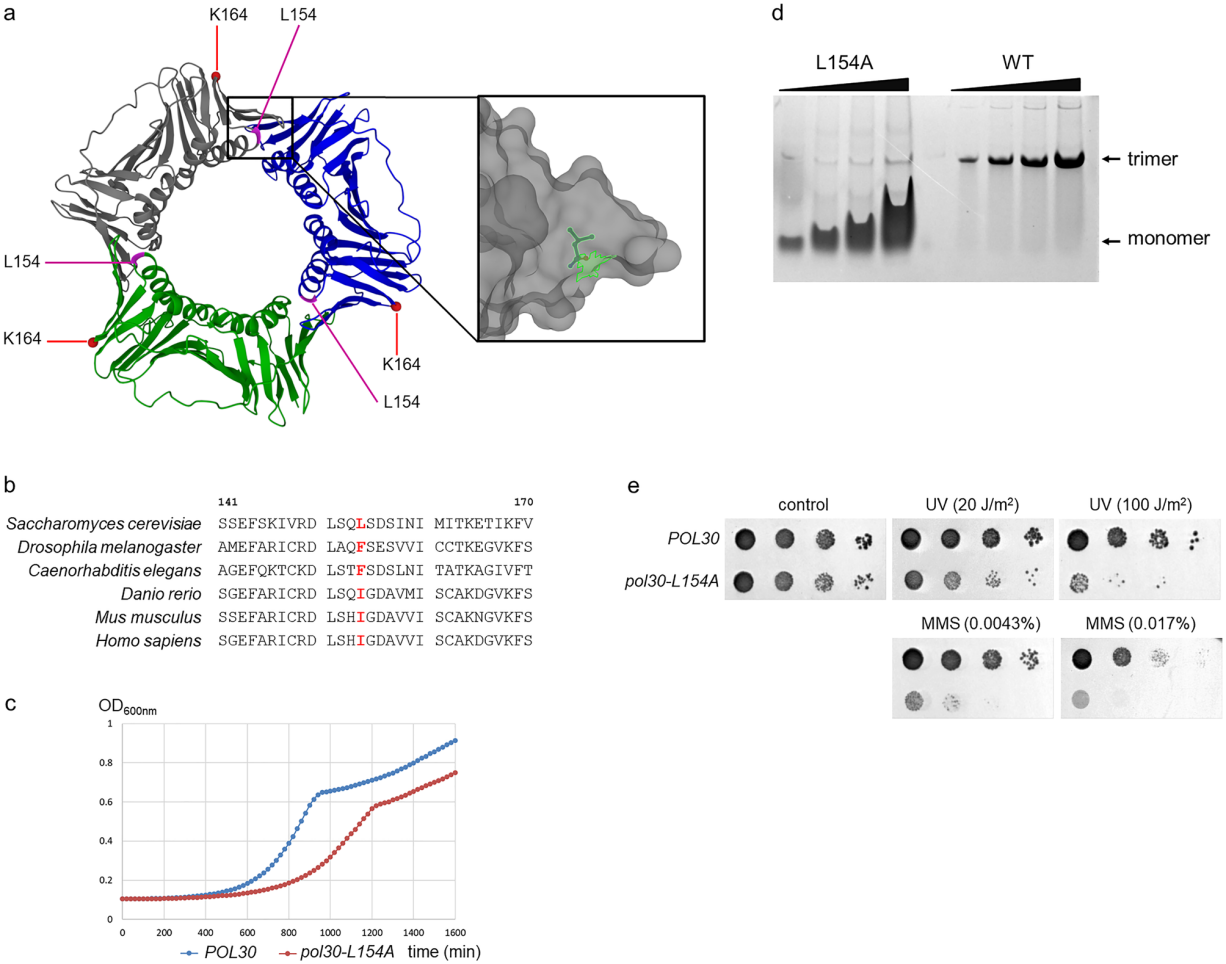
Characterization of *pol30-L154A*. (**a**) The position of the L154 amino acid is shown in a schematic ribbon diagram representing the three-dimensional structure of the PCNA trimer. The structure around L154 is shown in higher resolution. (source: Krishna, T.S.R et al. from RCSB PDB, PDB ID:1PLR)^6,62,63^. (**b**) Phylogenetic conservation of the residue at 154. The corresponding sequence from different species are shown and the residues at 154 are in red. Amino acid numbers are indicated at the top. In the case of C. elegans, the numbers are + 1 higher. (**c**) The pol30-L154A strain exhibits growth defects. The wild-type and L154A-PCNA expressing cells grew in YPD at 30 ˚C for 24 h and the optical density was recorded at every 20 min. The figure shows averages of three experiments each consisting of three technical parallels. (**d**) The L154A PCNA is a monomer in vitro. Increasing concentrations of purified wild-type and mutant PCNA were analyzed on a 12% non-denaturing polyacrylamide gel. (**e**) DNA damage sensitivity of pol30-L154A. Ten-fold serial dilutions of the indicated strains were spotted on media containing the indicated concentrations of MMS or irradiated with the indicated doses of UV.

We reported in a previous study the characterization of PCNA variants that we generated during a directed mutational screening of yeast *POL30*^32^. As part of that screen, we engineered a mutant in which the leucine at position 154 was replaced by alanine (L154A PCNA). The L154 amino acid is situated at the inner surface of the PCNA ring, at the end of an alpha helix (Fig. 1a). This position is not conserved evolutionally at the amino acid level, but the corresponding leucine, isoleucine, and phenylalanine residues found in different species all have hydrophobic sidechains (Fig. 1b). Because the hydrophobic sidechain points away from the center of the PCNA ring and forms part of a hydrophobic cavity (Fig. 1a), we surmised that the mutation would not affect the sliding of PCNA on DNA. Still, the *pol30-L154A* strain expressing the mutant PCNA from a yeast centromeric plasmid as a sole source of PCNA exhibited a discernible growth defect compared to the wild type (Fig. 1c). It raised the possibility, that due to the vicinity of L154 to the subunit interface, the mutation might affect trimer stability, thereby interfering with replication. Indeed, examination of the wild-type and mutant PCNA by non-denaturing polyacrylamide gel electrophoresis exposed the monomer nature of the mutant, whereas the wild type existed as a trimer in vitro (Fig. 1d). Additionally, further investigation revealed that the mutation also increased the sensitivity of the *pol30-L154A* strain to DNA damaging agents compared with the wild type (Fig. 1e). These results suggested that whereas the mutation influenced replication, it also compromised the activity of one or more DNA damage response (DDR) pathways.

### The L154A mutation in PCNA inactivates the *RAD6/RAD18*-dependent DNA damage tolerance

**Figure 2:**
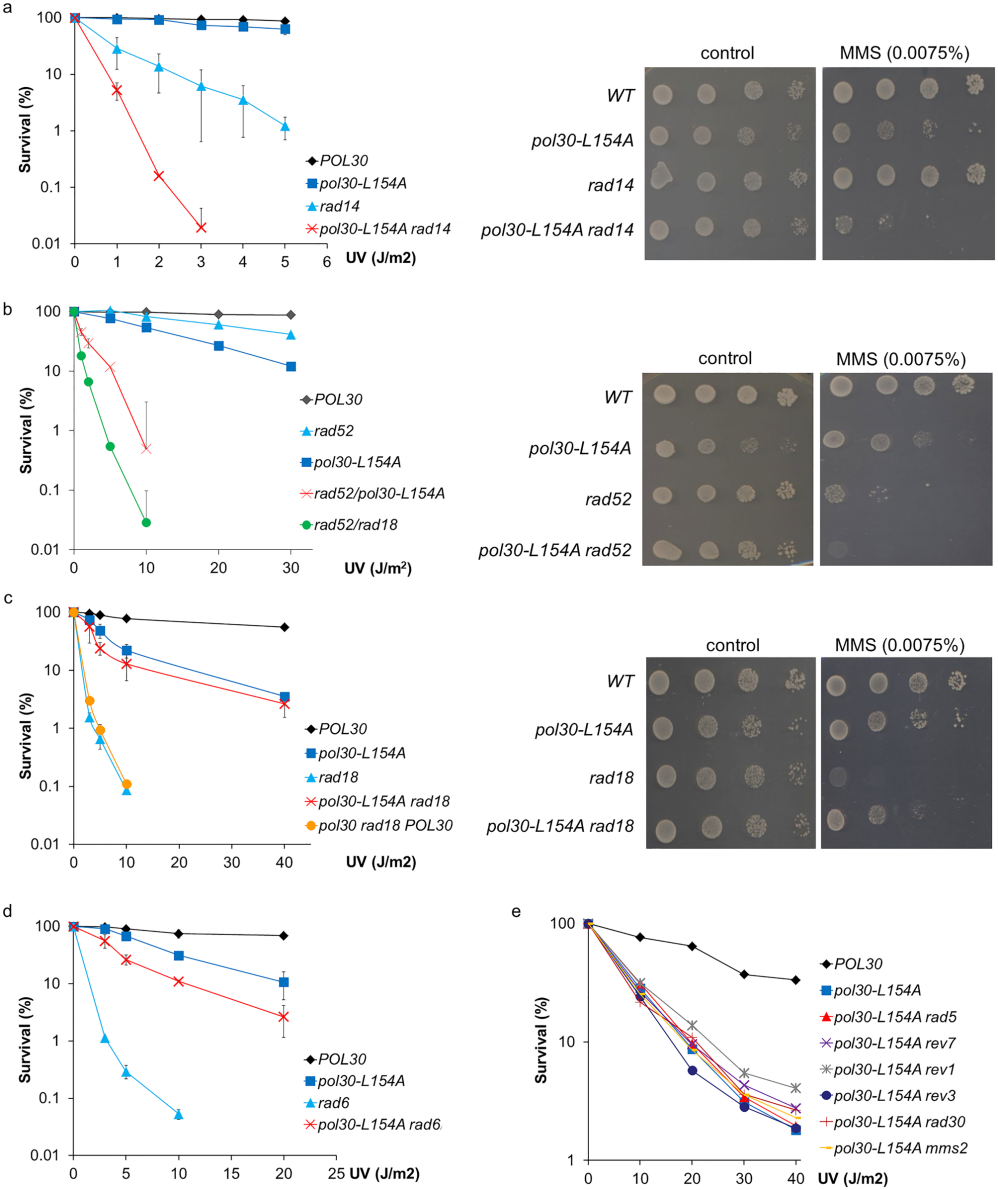
Genetic relation of *pol30-L154A* with DNA damage response pathways. (**a–c** left panels and **d–e**) Quantitative assay of UV-induced killing of the indicated strains. The results represent the average of three experiments. Standard deviations (SD) are indicated. (**a–c** right panels) Sensitivity of the strains to MMS. Ten-fold serial dilutions of the indicated strains were spotted on YPD plates containing the given amount of MMS.

First, we investigated the possible link between DDR and the PCNA mutation. Applying epistasis analysis, we found that neither nucleotide excision repair (NER) nor homologous recombination (HR) was compromised by the PCNA mutation, since deletion of *RAD14* or *RAD52*, the central regulators of NER and HR, respectively, in the *pol30-L154A* strain resulted in highly increased sensitivities upon both UV and MMS treatment, compared to the parental strains (Fig. 2a, b). Surprisingly, deleting *RAD18* governing DNA damage tolerance did not change the sensitivity of the *pol30-L154A* mutant (Fig. 2c). In other words, the high sensitivity of *rad18Δ* was suppressed to the sensitivity of the *pol30-L154A* strain. Moreover, the extreme sensitivity of *rad6Δ* was partially suppressed by the *pol30-L154A* mutation (Fig. 2d). To verify that the suppression was due to the PCNA mutation, we replaced the mutant PCNA expressing plasmid with one expressing the wild-type PCNA by plasmid shuffling in *rad18Δ*. Indeed, the expression of wild-type PCNA restored the high sensitivity of *rad18Δ*, confirming that the L154A amino acid change in PCNA alleviated the defect resulting from the absence of Rad18 (Fig. 2c). Testing several genes from the different sub-pathways of the Rad6/Rad18-governed DDR revealed that none of those genes could influence the DNA damage sensitivity of *pol30-L154A* (Fig. 2e). The results were confirmed using strains harboring *pol30-L154A* integrated into the *POL30* genomic locus. Based on these data, we concluded that the Rad6/Rad18-dependent DNA damage tolerance was not functional in the *pol30-L154A* strain.

### DNA damage-induced PCNA ubiquitylation and sumoylation at K164 are absent in *pol30-L154A*

**Figure 3:**
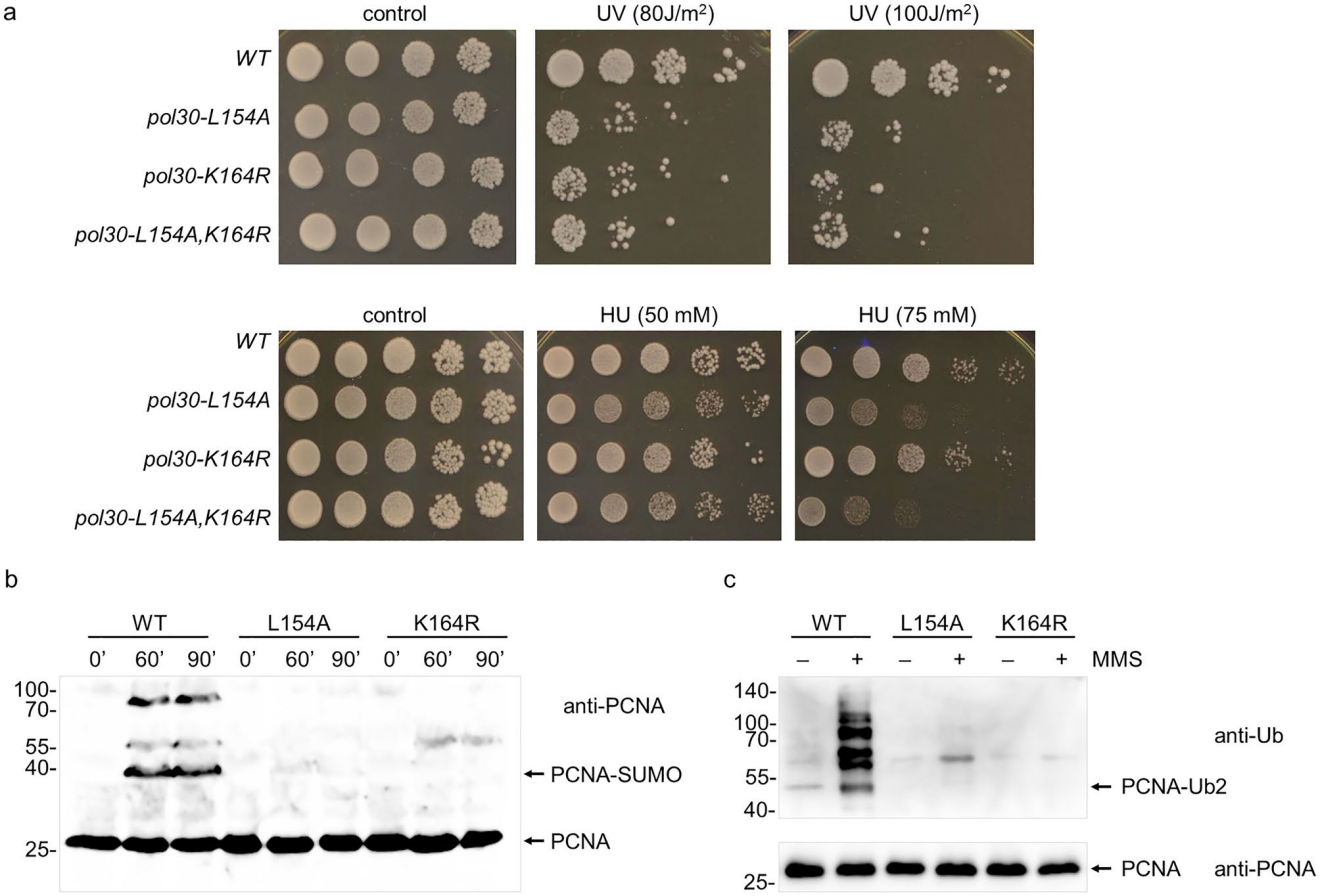
The L154A mutation prevents ubiquitylation and sumoylation at K164 of PCNA. (**a**) *pol30-L154A* and *pol30-K164R* are epistatic. Ten-fold serial dilutions of cells with the indicated genotypes were spotted on plates and exposed to the indicated doses of UV, or grown on medium containing HU at the indicated concentrations. (**b**) Sumoylation is absent in *pol30-L154A*. Strains expressing wild-type PCNA or its mutant variants were synchronized in G1 with α-factor and released back to cycling and treated with MMS for the indicated times. Unmodified and sumoylated PCNA were detected in whole-cell extract with an anti-PCNA antibody. (**c**). DNA damage-induced ubiquitylation cannot be detected in *pol30-L154A*. 7His-PCNA from whole-cell extracts, prepared after 2 h MMS treatment of G1 synchronized cultures, was bound to Ni-beads and ubiquitylated forms were detected using an anti-Ub antibody (upper panel). 10% of the input was run on a separate gel and the blot was probed with an anti-PCNA antibody (lower panel). Uncropped blot pictures are provided in the Supplementary Information file.

One well-characterized PCNA mutation is known from the literature that bestows a very similar phenotype. That is the *pol30-K164R* mutation that abolishes the lysine where DNA damage-induced PCNA monoubiquitylation by Rad16-Rad18 or sumoylation during replication by Siz1 and Ubc9 takes place^14^. The lack of both modifications results in a moderate sensitivity to DNA damaging agents since the Rad52-dependent homologous recombination, released from the inhibition of sumoylated PCNA, compensates for the sensitivity conferred by the inactivity of the Rad18 pathway^15^. We found that the *pol30-L154A* strain exhibited the same sensitivity to UV as the *pol30-K164R* strain (Fig. 3a). Moreover, the double mutant *pol30-L154A-K164R* strain was as sensitive as the single mutants. These results suggested that even the L154A mutation prevented both sumoylation and ubiquitylation of PCNA at K164. To corroborate this idea, we probed the DNA damage-induced ubiquitylation of PCNA in cells treated with 0.02% MMS. Though PCNA sumoylation occurs during normal replication, it is enhanced in response to sub-lethal doses of MMS^14^. Therefore, we also probed PCNA sumoylation in cells exposed to 0.3% MMS. As Fig. 3b, c shows, neither posttranslational modifications could be detected in the *pol30-L154A* or the *pol30-K164R* strain as opposed to the wild type demonstrating that the L154 residue is needed for both sumoylation and DNA damage-induced ubiquitylation of PCNA at K164.

### The L154 residue of PCNA mediates direct interaction with Rad18

**Figure 4:**
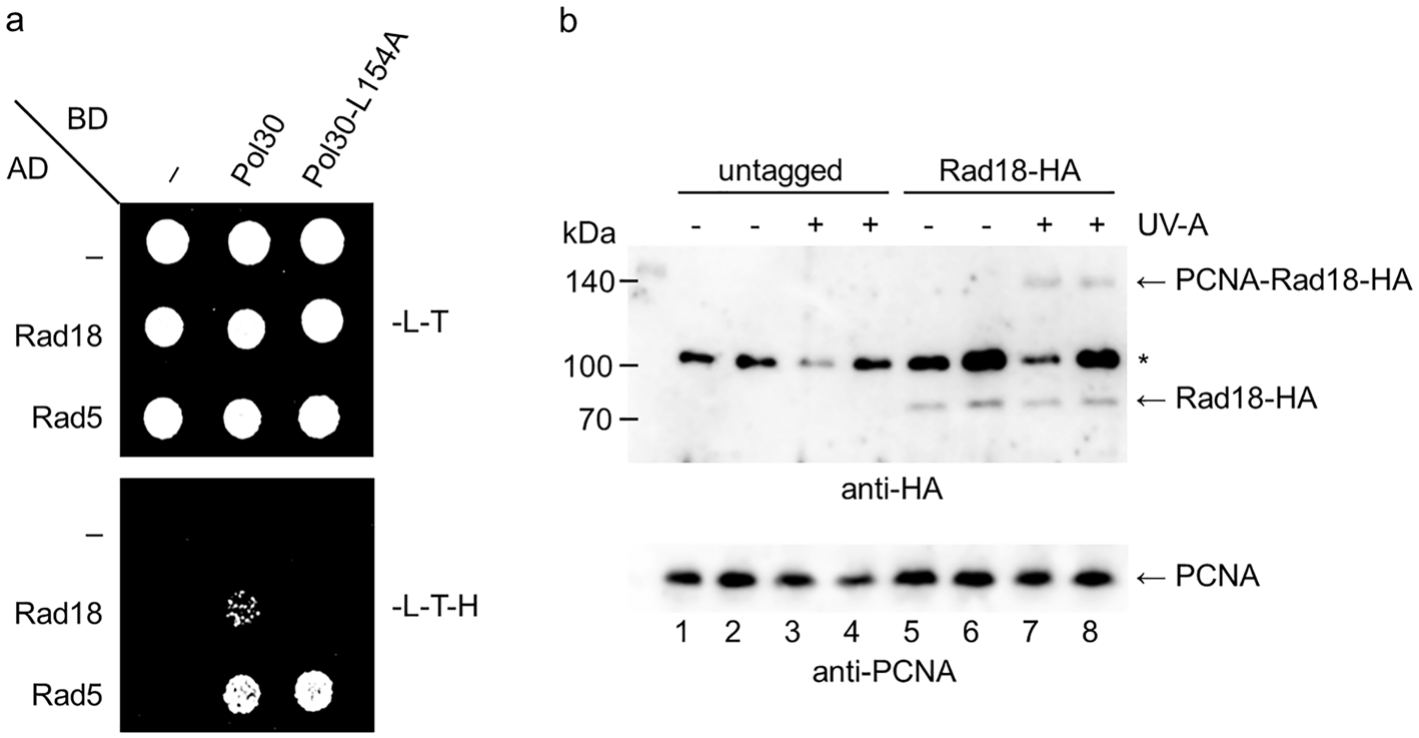
The L154 residue of PCNA is involved in interaction with Rad18. (**a**) The interactions between PCNA and Rad18 and Rad5 were tested by yeast two-hybrid assay. AD, fusion with Gal4 activation domain; BD, fusion with Gal4 DNA binding domain; –, empty vector; -L–T, -leu -trp control medium; -L–T-H, -leu-trp-his test medium. (**b**) Direct interaction of the L154 amino acid with Rad18 was examined in cross-linking experiments. Cells expressing PCNA-6His with an artificial amino acid at position 154 and Rad18 without or with a 3HA-tag were treated with UV-A to cross-link proteins contacting PCNA through the 154th residue. PCNA and its complexes were bound to Ni-beads, and the PCNA-Rad18 cross-linked product was visualized by an anti-HA antibody (upper panel). PCNA in the bound fractions were detected by an anti-PCNA antibody (lower panel). Two parallel samples are shown for each condition. * marks a non-specific band. Uncropped blot pictures are provided in the Supplementary Information file.

To get ubiquitylated at K164, PCNA has to interact with the ubiquitin ligase Rad18. We investigated the possibility that the L154A mutation hindered the binding of Rad18 to PCNA using yeast two-hybrid assays. Indeed, the L154A PCNA did not show interaction with Rad18, contrary to the wild type (Fig. 4a). In a control experiment, the L154A PCNA could still interact with Rad5, a known interacting partner of PCNA, indicating that the L154A mutation did not compromise the overall interaction capabilities of PCNA^14^. The role of the L154 amino acid in binding the SUMO ligase Siz1 could not be examined in similar assays as we could not detect the Siz1-PCNA interaction by yeast two-hybrid, in accord with a previous report (data not shown)^36^.

Whether the L154 residue was directly involved in Rad18-binding was assessed in in vivo cross-linking experiments based on the site-specific insertion of the UV-A-inducible cross-linker amino acid p-benzoyl-L-phenylalanine (pBPA)^37^. Due to the presence of phenylalanine at the corresponding position in flies and worms, we reasoned that the phenylalanine derivative pBPA would maintain the hydrophobicity of the surroundings therefore it could capture the interactions at the hydrophobic pocket. pBPA was inserted in the place of L154 in a plasmid-borne 7His-PCNA construct, and the presence of cross-linked Rad18-HA in purified PCNA fractions prepared from UV-A-treated or untreated cells was followed by a tag-specific antibody. As Fig. 4b shows, a Rad18-PCNA cross-linked product appeared in UV-A-irradiated samples indicating that the modified phenylalanine pBPA residue directly mediated the Rad18-PCNA interaction. This observation is in agreement with the results obtained by yeast two-hybrid and together they infer a direct role for the L154 of PCNA in binding Rad18. However, as the experiment was carried out in the absence of any DNA damaging treatment, we could not conclude whether Rad18 was bound to free PCNA or to PCNA loaded on DNA.

### The L154A PCNA mutation affects replication

**Figure 5:**
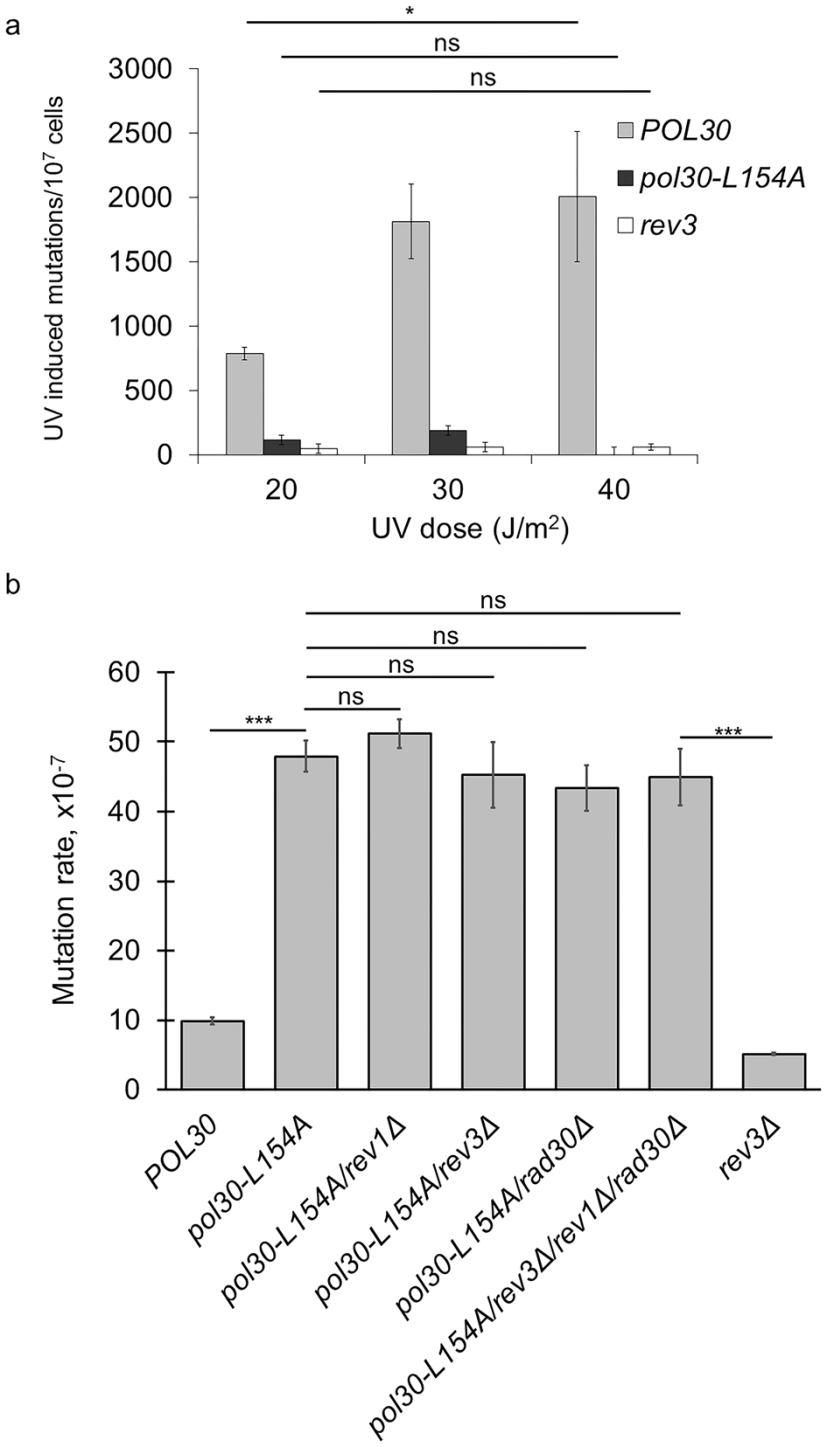
Mutagenesis in *pol30-L154A*. (**a**) Mutagenesis is not induced by UV in *pol30-L154A*. Forward mutation rates at the *can1* locus were determined in strains irradiated with the indicated doses. Mutations occuring spontaneously were subtracted from the total number of mutations. (**b**) The *pol30-L154A* strain exhibits high spontaneous mutation rate independent of the TLS polymerases. Forward mutation rates at the *can1* locus were determined using 10 parallel cultures. The results represent the averages of 4 experiments. SD is also shown. P-values representing the significance of difference are shown as *p < 0.05; **p < 0.01; ***p < 0.001.

Several mutations had been identified at the PCNA subunit interface which caused trimer instability as judged by the monomeric behavior in vitro, but still supported normal cellular growth suggesting that in vivo the mutants formed a trimer during replication^38–40^. It applied that the trimer instability observed with the L154A PCNA in vitro was not necessarily the cause of the observed replication defect of the mutant strain. Therefore, we set out to further characterize the effect of *pol30-L154A* on replication. The *pol30-L154A* strain exhibited marked sensitivity to the replication inhibitor hydroxyl-urea (HU) (Fig. 3a). Because the *pol30-K164R* strain devoid of sumoylation and DNA damage-induced PCNA ubiquitylation was as sensitive as the wild type, and the double mutant *pol30-L154A*-*K164R* strain showed the same HU-sensitivity as the *pol30-L154A* single mutant, we concluded that the influence on replication was independent of the posttranslational modifications of PCNA at K164. To further characterize its replication deficiency, we measured mutagenesis in the mutant strain. As expected from the inactivity of the *RAD18* pathway, induced mutagenesis was abolished in the *pol30-L154A* strain (Fig. 5a). Importantly, though, spontaneous mutagenesis was ~ 5-fold higher in the *pol30-L154A* strain as compared to the wild type (Fig. 5b)^21^. To examine the involvement of TLS polymerases, we tested the effects of the single gene deletions of *REV3, REV1,* and *RAD30* in the *pol30-L154A* strain. However, none of the deletions affected the rate of spontaneous mutations, and even the *pol30-L154A rev3Δ rev1Δ rad30Δ* quadruple mutant exhibited the same high mutation rate as the *pol30-L154A* strain. These observations demonstrated that the elevated spontaneous mutation rate in the *pol30-L154A* strain was independent of the TLS DNA polymerases.

### Polymerase slippage frequently occurs in the *pol30-L154A* strain

**Figure 6:**
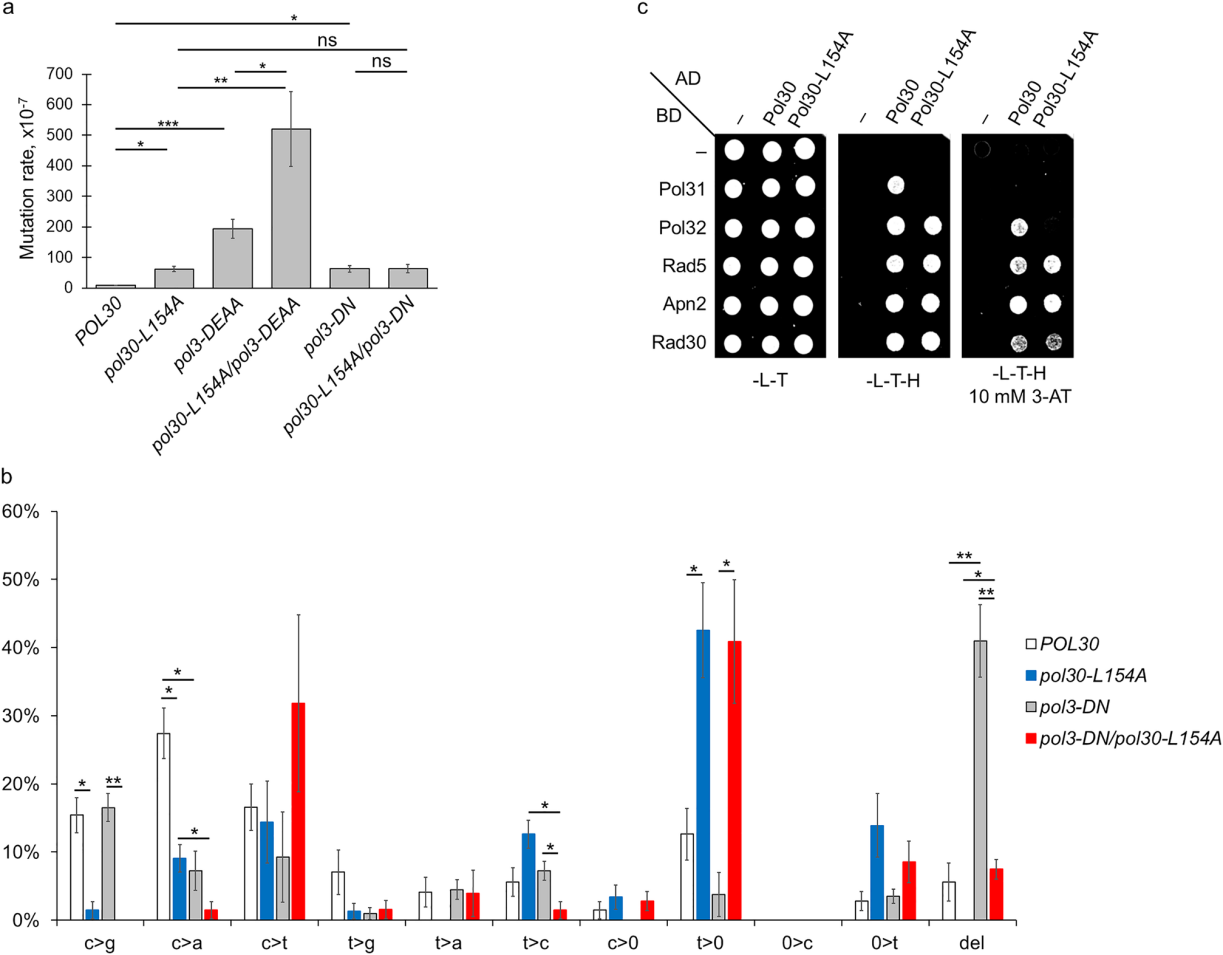
The PCNA-Polδ complex is affected in *pol30-L154A*. (**a**) The *pol30-L154A* mutation is epistatic to the *pol3-D643N* polymerase domain mutant. Spontaneous forward mutation rates at the *can1* locus were measured using 10 parallel cultures for each strain. The results represent the averages of 4–12 experiments. (**b**) Spontaneous mutation signature of *pol30-L154A*. The *can1* gene from independent colonies arising in the spontaneous mutagenesis experiments was sequenced and mutations were classified into categories indicated on the x axis. Average mutation frequency and SD of each mutation category were calculated from 64 (*POL30*) or 72 (*pol30-L154A*) independent mutational events, from 4 experiments. (**c**) Binding of the Polδ subunits Pol31 and Pol32 to PCNA is weakened by the L154A mutation, as examined by yeast two-hybrid. Labelling as in Fig. 4.

Our results suggested that the replicative polymerase was responsible for generating excess spontaneous mutations in the *pol30-L154A* strain. Therefore, we investigated which activity of Polδ was affected by the L154A PCNA by generating genomic mutations in the Polδ exonuclease and polymerase domains, separately, and comparing the rate of spontaneous mutagenesis of the single mutants to double-mutant strains carrying the *pol30-L154A* mutation, additionally. As Fig. 6a shows, the D321A E323A amino acid changes in the Pol3 exonuclease active site (*pol3-01*) increased the rate of spontaneous mutations ~ 20-fold compared to the wild type^41,42^. However, in the presence of the *pol30-L154A* allele, the mutation rate was further elevated to ~ 50-fold. On the other hand, the D643N mutation in the polymerase domain (*pol3-t*) caused a ~ 5-fold increase in the mutation rate, which remained at the same level even in the *pol30-L154A pol3-D643N* double mutant strain^42,43^. These data showing a synergistic relationship between *pol30-L154A* and *pol3-DE321,323AA*, and epistasis between *pol30-L154A* and *pol3-D643N* revealed, that the observed increase in spontaneous mutagenesis was due to replication errors linked to the polymerase activity of Polδ, whereas the exonuclease activity of the enzyme was not affected by the PCNA mutant.

The *pol3-D643N* mutation induces frameshifts in homonucleotide runs and repeat-associated extended deletions due to replication slippage^42^. To identify the mutations in the *pol30-L154A* strain, we sequenced the whole *CAN1* gene from mutant colonies grown on canavanine-containing plates. As Fig. 6b shows, the most prevalent change detected in *pol30-L154A* was single-nucleotide deletion at short homonucleotide T or A runs accounting for more than 40% of the mutations, in contrast to the ~ 10% observed with the wild type. Additionally, ~ 15% of the mutations were single-nucleotide insertions in the mutant as opposed to the ~ 3% in the wild type. Meanwhile, C to G mutations, generally considered to be produced by the TLS DNA polymerase Rev1, dropped from ~ 15% found in the wild type to ~ 1% in the mutant. Together, these results suggested that misalignment events at the primer end provoked replication slippage in the *pol30-L154A* strain resulting in one nucleotide deletions/insertions at short homonucleotide runs. The mutation spectrum of the Polδ mutant *pol3-D643N* exhibited a striking difference from that of *pol30-L154A* in that single nucleotide deletions accounted for only a few percent of the mutations, whereas extended deletions with a median length of ~ 80 nucleotides caused more than 40% of spontaneous mutations. Importantly, the double mutant *pol3-D643N pol30-L154A* behaved like *pol30-L154A* in that the ratio of single nucleotide deletions was as high as in *pol30-L154A*, whereas the ratio of extended deletions dropped back to a few percent of the wild-type level. It indicated that the *pol30-L154A* mutation was dominant over the *pol3-D643N* mutation.

### The Polδ-PCNA complex is altered in the *pol30-L154A* strain

The above results suggested that the stability of the replication complex was extenuated in *pol30-L154A.* This prompted us to examine the interaction between PCNA and Polδ. Polδ consists of three peptides. Pol3 is the catalytic subunit, containing both the polymerase and exonuclease active sites. Pol31 is essential, whereas Pol32 is a non-essential accessory subunit^44^. All three subunits of Polδ have been shown to contain sequence motifs resembling the conserved PIP motif, and mutating any of them decreased DNA synthesis by Polδ in vitro in the presence of PCNA, RFC, and RPA^45^. Our experiments, testing the interactions of the three subunits of Polδ by yeast two-hybrid, revealed that Pol32 interacted with both the wild-type and the L154A PCNA, though the interaction with the mutant was weakened as evidenced by the lack of growth of the corresponding strain on 3-AT containing medium that provides more stringent selection for interactions (Fig. 6c). Moreover, Pol31 did not show interaction with the mutant PCNA, only with the wild type. In contrast, association with Rad5, Polη, and Apn2 were not affected^9,14,46^. We note, that the Pol3-PCNA interaction could not be examined in these assays as there was no detectable interaction even when using the wild-type PCNA construct. In summary, these results suggested that the L154A PCNA mutation weakened the interaction of the Polδ with PCNA.

## Discussion

In this study, we have identified a PCNA residue that is essential for both DNA damage tolerance and normal replication through mediating interactions with key proteins of the two processes.

The L154A PCNA mutation is situated at the inner side of PCNA, on the front of the molecule. It confers yeasts growth defect, HU sensitivity, and elevated spontaneous mutagenesis resulting from increased rates of single-nucleotide insertions and deletions at homopolymer nucleotide runs, all indicating difficulties during replication. The mutation causes trimer instability in vitro probably due to its proximity to the interdomain surface, which might lie behind the observed in vivo replication defects. However, this conclusion is contradicted by several PCNA mutations that render PCNA monomer in vitro, but do not lead to phenotypes associated with replication errors, when expressed in yeast cells. For example, the PCNA protein with the G178S amino acid change at the subunit interface is a monomer in vitro, but the corresponding yeast strain grows at the wild-type rate^39,40^. The same is true for the *pol30-41* strain having the A112T and S135F replacements in PCNA^38^. In addition, *pol30-41* exhibits a wild-type rate of spontaneous mutagenesis. These data indicate that the inability of PCNA to form trimers in vitro does not necessarily manifest in vivo. In any case, our experiments showing decreased binding of Pol31 and Pol32 to L154A PCNA suggest, that the replication defect of the mutant is caused by faulty interaction between PCNA and Polδ. Recent studies reported that only the catalytic subunit of Polδ interacts with PCNA and the interaction is mediated by the CysA element of Pol3 containing a Zn-finger and a PIP motif, whereas the regulatory subunits are positioned away from the PCNA ring^47–49^. The crystal structure of Polδ with PCNA and DNA also reveals that the amino acids involved in Pol3-binding are situated at the IDCL of PCNA, which is far from the L154 residue. In light of these, it is interesting that Pol32 could interact with the wild-type and though with decreased affinity also with the mutant PCNA in yeast two-hybrid, whereas Pol31 showed interaction only with wild-type PCNA. As the PIP motif at the C-terminus of Pol32 was shown to bind PCNA in isothermal calorimetric experiments and yeast two-hybrid, the interaction we observed is probably direct^50,51^. On the other hand, the interaction of Pol31 with PCNA could be indirect, mediated by endogenous Pol3. In either case, as in yeast two-hybrid PCNA can probably not form a trimer due to its fusion to the Gal4 activation domain, we can rule out that trimer instability affected the interactions. We surmise, that the L154 PCNA residue might directly bind Polδ and its role would be to help correct positioning of the polymerase during the establishment of the DNA-PCNA-Polδ complex. Hence, in its absence, an erratic complex is formed, in which Polδ is prone to dis-attach from the primer end.

Interestingly, the L154 residue is also directly involved in Rad18 binding, as we show by yeast two-hybrid and direct in vivo cross-linking experiments. The failure of Rad18 to interact with L154A PCNA prevents DNA damage-induced PCNA ubiquitylation, evidenced by the lack of ubiquitylated PCNA upon MMS treatment. Accordingly, the Rad18-governed DDT is completely inactive in *pol30-L154A*. Nevertheless, the *pol30-L154A* strain is much more resistant to DNA damaging agents than the *rad18* strain. Its equal sensitivity to and epistatic relationship with *pol30-K164R*, together with the lack of sumoylated PCNA in cells indicate that the L154A mutation prevents PCNA sumoylation at K164, as well, though we could not detect a direct Siz1-PCNA interaction using in vivo cross-linking. However, an important difference between *pol30-L154A* and *pol30-K164R* is that they exhibit differing UV sensitivities in *rad52Δ* background. The *pol30-K164R rad52Δ* double mutant is as sensitive as the *rad6Δ rad52Δ* strain indicating that HR released from the suppression of sumoylated PCNA compensates for the inactivity of the *RAD6* pathway in *pol30-K164R* ^15^. In contrast, we have found, that the *pol30-L154A rad52Δ* strain is less sensitive than the *rad18Δ rad52Δ* mutant suggesting that a DNA damage tolerance mechanism is activated in *pol30-L154A rad52Δ* cells.

**Figure 7:**
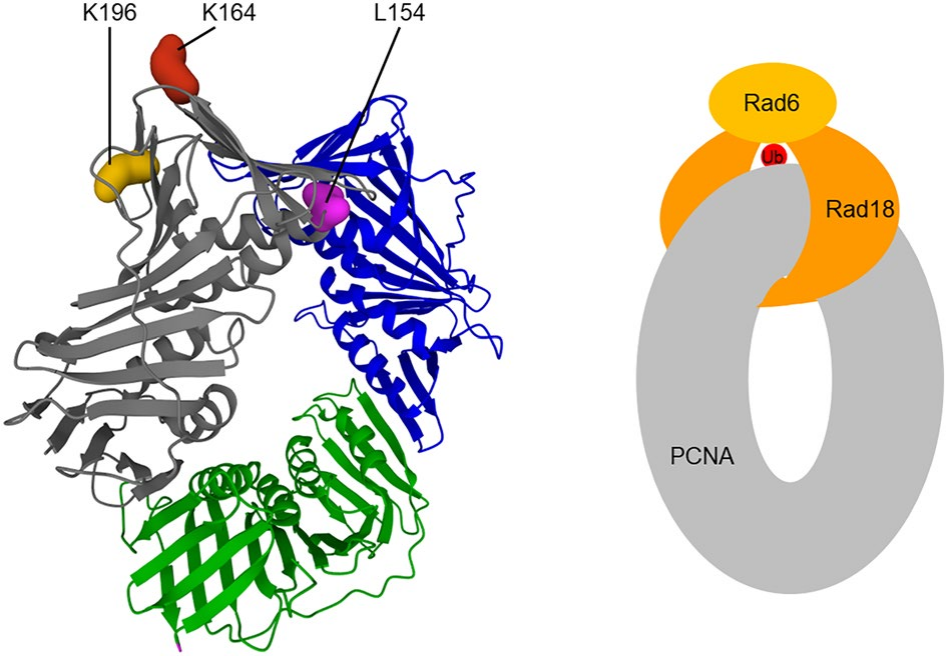
Model of the Rad18-PCNA complex. In the left, the positions of L154 in the front face and K196 on the back face of PCNA are shown, together with K164. On the right, the schematic representation of the model is depicted.

We note, that the K196V mutation of yeast PCNA was described as defective in the interaction with Rad18, and it bestowed the same sensitivity to yeasts as the K164R PCNA mutation^52^. Based on this, we infer that sumoylation at K164 is also hindered in the *pol30-K196V* mutant. This, together with our findings suggest that Rad18 and a sumoylation enzyme, probably Siz1, connect PCNA through the same residues, this way ensuring the mutually exclusive ubiquitylation and sumoylation at K164. Curiously, closer examination of the positions of the K196, L154, and K164 residues in the crystal structure of PCNA revealed that they could be connected through a circle around the PCNA subunit. Using this information and also taking into consideration previous results showing that Rad18 works as a dimer which binds one Rad6 molecule, but the dimerization does not involve the Zn-finger of Rad18 and that the PCNA interacting surface was mapped to almost all parts of human Rad18, we set up a model of the PCNA-Rad18 interaction (Fig. 7)^53–55^. In this model, two Rad18 molecules form a circle around PCNA by binding each other through the RING domains at the inner side of the PCNA ring, whereas the Rad6-binding sites are at the outer surface of PCNA positioning Rad6 close to K164.

In summary, we surmise that the L154 PCNA residue forms part of a hydrophobic pocket mediating a direct interaction with Rad18 and probably with Polδ, which is abrogated by the lack of the hydrophobic side chain caused by the L154A mutation. We also conclude that the composite influence of the L154 amino acid of PCNA on genome stability foreshadows a regulatory mechanism that harmonizes replication and DNA damage response and demonstrates an emerging complexity of interactions mediated by the PCNA inner surface.

## Methods

### Yeast strains and plasmids

Yeast strains used in this study are derivatives of EMY74.7 (*MAT*a, *his3*-Δ*1*, *leu2-3*, -*112*, *trp1*Δ, *ura3-52*). Deletions were constructed by gene replacement. Point mutations were generated by a PCR-based method according to the Stratagene “Quick Change Site Directed Mutagenesis” protocol. For the genetic studies, the wild-type and the mutant *POL30* alleles with the promoter and terminator sequences were cloned into the centromeric low-copy plasmid YCplac33 (wt:pID1260, L154A PCNA:pID181, K164R PCNA:pID170). To check in vivo ubiquitylation, the *pol30* genes were N-terminally tagged with 7His by PCR resulting in pID1243 (7His-PCNA), pID1254 (7His-L154A PCNA), and pID1255 (7His-K164R PCNA). The whole sequence of each allele was confirmed by sequencing. A *pol30Δ, bar1Δ* derivative of the DF5a strain (*MATa, trp1-1, his3–200, ura3-52, lys2-801, leu2-3,2–112*)^56^ ectopically expressing one of the 7His-PCNA variants was applied to investigate the posttranslational modification of PCNA. For in vivo UV-A induced protein cross-linking, a BJ5464 strain (*MATα ura3-52 trp1 leu2d1 his3-D200 pep4::HIS3 prb1D1.6R can1 GAL*) (ATCC stock centre) derivative expressing C-terminally 3xHA-tagged Rad18 from the genomic locus was used. The strain was transformed with a 2µ based high-copy number pESC vector expressing the artificial amino-acil synthetase and the artificial tRNA^57^, and with another 2µ based high-copy number p426 plasmid expressing C-terminally 6xHis-tagged *pol30-L154TAG* from the GPD promoter (pID1222). For the two-hybrid assays, the yeast strain PJ69-4A (*MAT*a *trp1-901 leu2-3,112 ura3-52 his3-200 gal4Δ gal80Δ MET2:GAL7-lacZ LYS2:GAL1-HIS3 GAL2-ADE2*) was used^58^. The coding regions of *APN2*, *RAD5, POL31, POL32*, wild-type, and mutant *POL30* alleles were cloned into the Gal4-binding domain fusion pGBT9 vector resulting in pID1159, pID623, pID1023, pID1138, pID998, and pID1005, respectively. *RAD5*, *RAD18*, *POL30*, and *L154A pol30* were cloned into the Gal4 activation domain fusion vector pGAD424 + 1 resulting in pID145, pID684, pID999, and pID1004, respectively. The DE321,323AA exonuclease and D643N polymerase domain mutations in *POL3* were made using a CRISPR-Cas9 system^59^. To facilitate protein purification, the open reading frames of *pol30* alleles were cloned in N-terminal fusion with GST and expressed under the control of a galactose inducible phosphoglycerate promoter (pBJ842 backbone) in the protease deficient yeast strain BJ5464 .

### Sensitivity assays

For qualitative assays, cells were grown in YPD overnight at 30 ℃. Cells were counted using a Bürker chamber, then cultures were adjusted to the same density and a series of tenfold dilutions of the cultures were spotted onto plates. For UV sensitivity, plates were exposed to different doses of UV (254 nm) radiation and incubated at 30 ℃ in the dark for 3 days. Methyl methanesulfonate (MMS) and hydroxyurea (HU) sensitivities were assayed on plates supplemented with the indicated concentrations of the chemicals (Sigma-Aldrich).

For quantification of UV sensitivity, cells were spread onto YPD plates at appropriate dilutions and irradiated with UV for given times to apply the required dosages. Colonies were counted after incubating the plates in the dark at 30 ℃ for 3–5 days.

### UV-induced and spontaneous mutation rates

UV irradiation-induced forward mutation frequencies at the *CAN1* locus were measured by comparing the numbers of *can1*^*R*^ colonies at different UV doses, selected on synthetic complete (SC) -arg plates containing canavanine, with the number of colonies on SC plates exposed to the same UV doses. Spontaneous forward mutation frequencies were measured at the *CAN1* locus. Ten single colonies for each strain were diluted in water. Approximately 10 cells from each colony were inoculated into 500 µl SC in a microtiter plate and grown for 3 days at 25 °C that is the permissive temperature for the *pol3-t* mutants. The cultures were plated onto canavanine containing SC-arg plates, and the average number of mutants in the case of each strain was counted for 10^7^ plated cells. Mutation frequencies were determined using a chart based on the Lea-Coulson fluctuation model.

### Sequencing of the *CAN1* gene

For sequencing of the *CAN1* gene, 3 canavanine resistant single colonies were picked from each plate of the spontaneous mutagenesis experiment and grown in 2 ml YPD overnight. Altogether, 84–84 colonies from the wild-type and the mutant strains were examined. Genomic DNA was isolated from the cultures, and the *can1* gene was amplified with 5’-GAAGAGTGGTTGCGAACAGAG-3’ and 5’-CTTATGAGGGTGAGAATGCG-3’ oligonucleotides. The PCR products were sequenced using the 5’-CTGTCACGCAGTCCTTG-3’, 5’-GGTGAGAATGCGAAATGG-3’ and 5’-ATACTAATCCATGCCGCC-3’ gene specific primers.

### Growth assays

To compare the growth of the strains, a Synergy 2 automated microplate reader machine (BioTek, USA) was used. Aliquots of 1 μl of overnight starter cultures of the wild-type and the mutant *pol30* expressing strains were transferred into 100 μl of fresh YPD in dedicated 96-well plates. Absorbance at 600 nm was measured every 20 min for 24 h at 30 °C with continuous shaking. Averages of 3 experiments with 3–3 technical parallels in each are shown.

### Yeast two-hybrid

The binding and activation domain fusion constructs were co-transformed into PJ69-4A^58^, and transformants were selected on SC-leu/trp medium. Single colonies of the transformants were resuspended in water at equal densities and spotted onto an SC-leu/trp plates to demonstrate growth and onto an SC-leu/trp/his plates to assess the *HIS3* gene expression reflecting protein–protein interaction. 3-amino-triazole (3-AT) was added to SC-leu/trp/his plates at defined concentrations to test the strength of the interactions. Plates were incubated at 30 ℃ for 3–5 days.

### In vivo PCNA ubiquitylation and sumoylation

PCNA ubiquitylation was detected by applying denaturing Ni-nitriloacetic acid (Ni–NTA) chromatography^60^. Briefly, 200 ml yeast cultures grown in YPD to A_600_:0.8 were synchronized by alpha-factor for 3.5 h. After microscopic verification of the G1 state of the cultures, they were released back to cycling in fresh YPD, and half of the cultures were treated with 0.02% MMS for 90 min. After harvesting, cell pellets were frozen in liquid nitrogen. Following cell lysis in cold 1.85 N NaOH, 7.5% β-mercaptoethanol, proteins were precipitated by trichloroacetic acid (TCA) and resuspended in Buffer A (6 M guanidine HCl, 100 mM sodium phosphate, 10 mM Tris/HCl, pH 8.0) by rotating for 1 h at room temperature. Lysates were clarified and adjusted to pH ~ 8 then samples were bound to Ni–NTA agarose in the presence of 0.05% Tween-20 and 10 mM imidazole overnight. After binding, beads were washed with Buffer A and Buffer B (8 M urea, 100 mM sodium phosphate, 10 mM Tris, pH 6.3, 0.05% Tween-20), and bound proteins were eluted for 10 min at 60 °C in 20 µl of buffer C (8 M urea, 200 mM Tris/HCl, pH:6.8, 1 mM EDTA, 5% SDS, 0.1% bromophenol blue, and 1.5% DTT), and proteins were resolved on a 12% SDS-PAGE. Following blotting, the PVDF membrane (Immobilon-P, Merck Millipore Ltd.) was pretreated in 6 M guanidyl-HCl, 20 mM Tris/HCl pH:7.5, 5 mM 2-ME for 30 min at 4 °C to probe with anti-Ub antibody (2000×, Santa Cruz sc8017). An aliquot of the same samples was run on a second gel that was developed by a PCNA antibody. Anti-mouse (30,000×, Thermo Scientific 31430) and anti-rabbit (10,000×, Thermo Scientific 31460) secondary antibodies were applied.

PCNA sumoylation was detected in whole-cell extracts. 15 ml of fresh cultures were grown in YPD to OD 0.7–0.8, synchronized by alpha-factor for 2 h, and treated with 0.3% MMS. At given time points 2 ml aliquots were collected, and whole-cell extracts were prepared using NaOH/TCA^60^. Pellets were resuspended in buffer C, and after boiling, proteins were resolved on a 12% SDS-PAGE. PCNA was visualized using anti-PCNA primary (20,000×) and anti-rabbit (10,000×, Thermo Scientific 31460) secondary antibodies.

### In vivo UV-A induced protein cross-linking

3 ml yeast cultures were incubated overnight in SC-ura/trp to select for the PCNA-6His and the artificial amino-acil synthetase expressing constructs. The next day cultures were diluted in 50 ml fresh media containing 1 mM pBPA (p-benzoyl-L-phenylalanine), and further incubated overnight. Cultures were diluted to OD_600_: 0.15 in 50 ml fresh media. After 1.5 h of incubation, cells were collected, washed, and resuspended in 10 ml PBS. Half of the cultures were irradiated with UV-A (365 nm) for 30 min on ice in a Petri dish with constant shaking. Cells were broken as described earlier^61^. Proteins were eluted from the Ni-beads by boiling and resolved on an 8 and a 10% SDS-PAGE. Rad18 cross-linked to PCNA was visualized using anti-HA (10,000×, Abcam ab9110) and anti-rabbit (10,000×, Thermo Scientific 31460) antibodies. To detect PCNA the membrane was developed with anti-PCNA (5000×) and anti-rabbit (10,000×, Thermo Scientific 31460) antibodies.

### Statistical analysis

Student’s t-test using Excel (Microsoft, Redmond, WA, USA) was applied to compare separate groups. *P*-values of < 0.05 were considered statistically significant. *p < 0.05, **p < 0.01, ***p < 0.001.

## Supplementary Information

Supplementary Information.
