# Supplementary material for: The inner side of yeast PCNA contributes to genome stability by mediating interactions with Rad18 and the replicative DNA polymerase δ
Source: Sci Rep. 2022 Mar 25;12:5163. doi: 10.1038/s41598-022-09208-7 (PMC8956578; doi:10.1038/s41598-022-09208-7)
Supplement: Supplementary file 1 — Supplementary Information. [file 41598_2022_9208_MOESM1_ESM.docx]

**The inner side of yeast PCNA contributes to genome stability by mediating interactions with Rad18 and the replicative DNA polymerase δ**

Robert Toth ^†^, Miklos Halmai ^†^, Zsuzsanna Gyorfy, Eva Balint and Ildiko Unk*

Supplementary files

WT

L154A


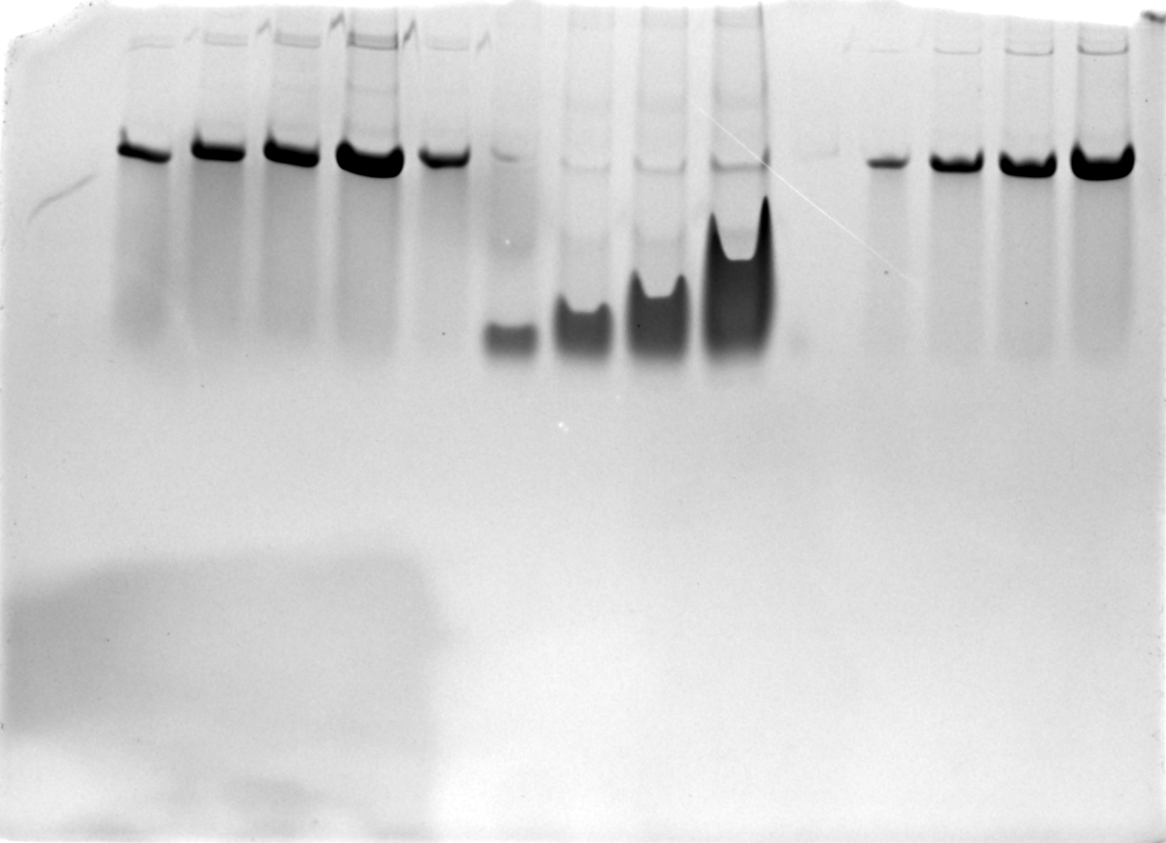


Fig. S1. Full size image of the blot corresponding to Fig. 1d. The portion of the gel used for the figure is outlined with red lines.

K164R

L154A

WT

0’ 60’ 90’ 0’ 60’ 90’ 0’ 60’ 90’


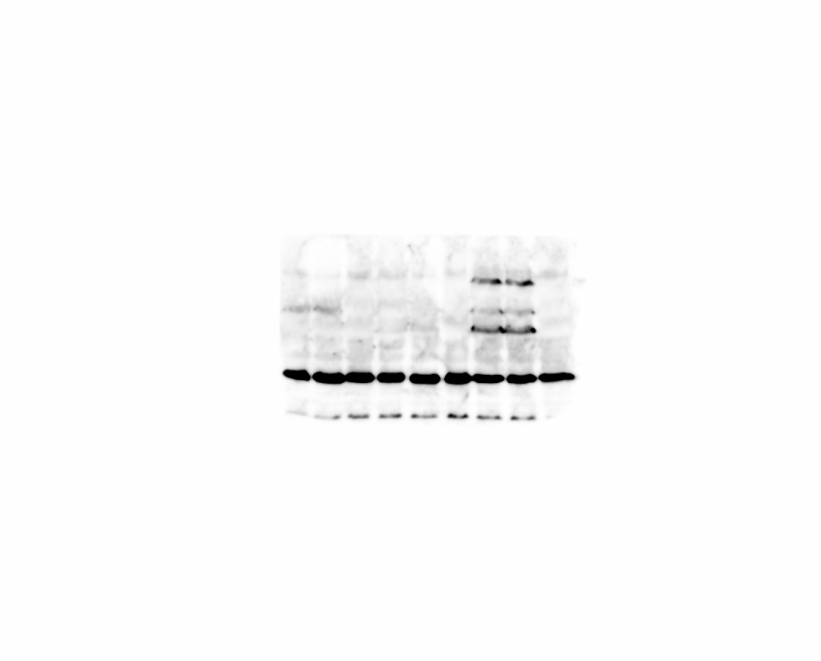


Fig. S2. Full size image of the blot corresponding to Fig. 3b. The edges of the blot are outlined with solid black line.

WT

L154A

K164R

– + – + – + MMS


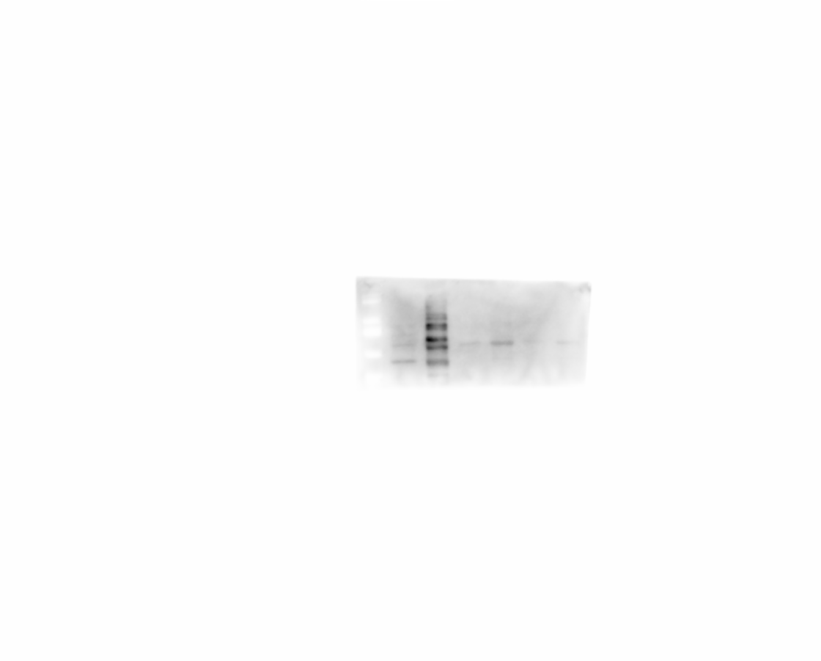


K164R

L154A

WT

K164R

L154A

WT

– + – + – + MMS

– + – + – + MMS


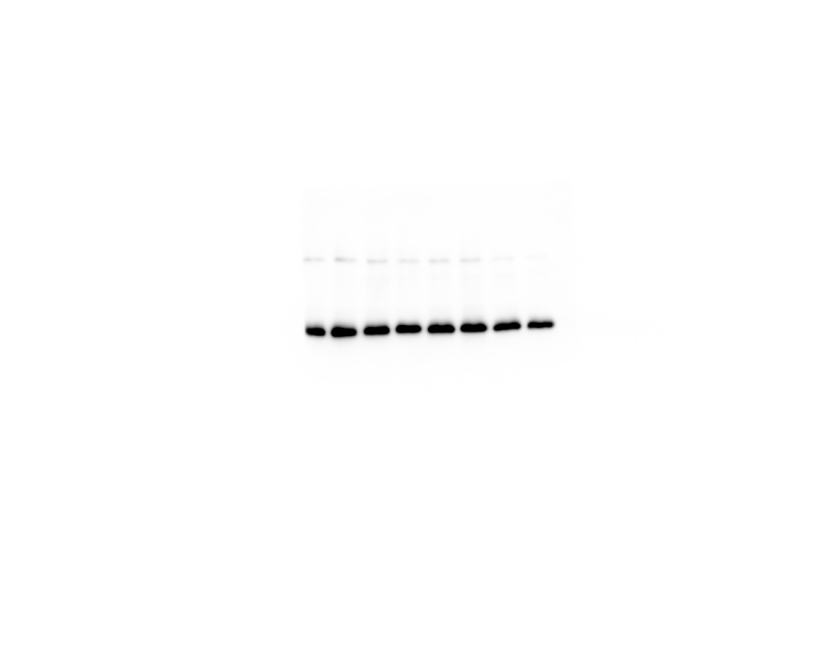

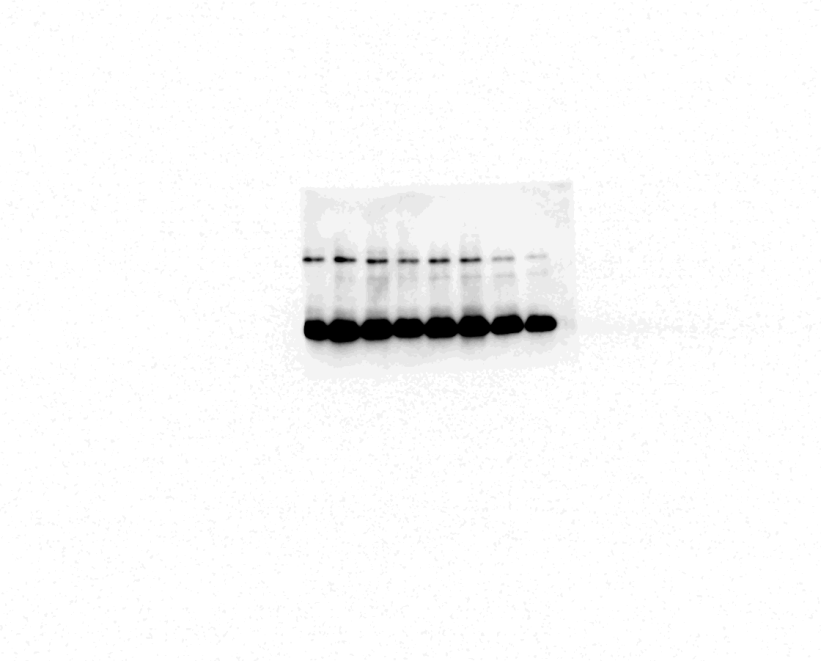


Fig. S3. Full size images of the blots corresponding to Fig. 3c. The edges of the blots are outlined with solid black lines, while the portion of the gel used for the figure is outlined with red lines. A lighter and a darker exposure of the blot is shown for the bottom part of Fig. 3c.

anti-HA

untagged Rad18-HA

- - + + - - + + UV-A


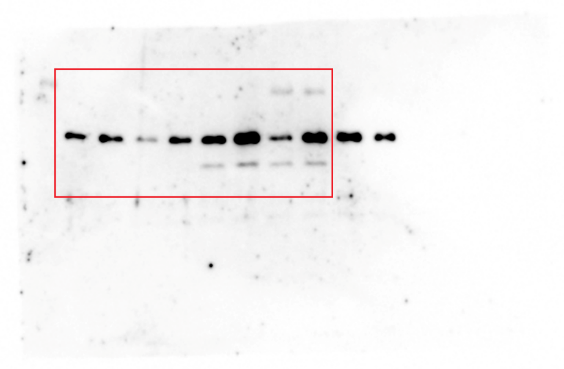


anti-PCNA

untagged Rad18-HA

- - + + - - + + UV-A


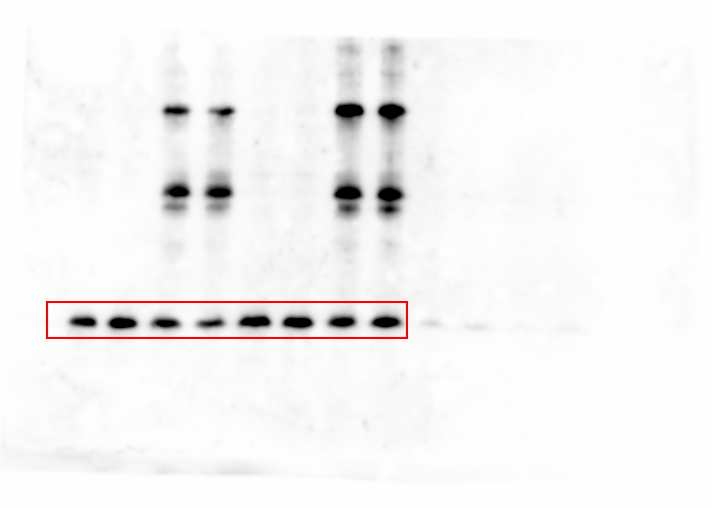


← PCNA-6His

PCNA-

crosslinked

products

Fig. S4. Full size images of the blots corresponding to Fig. 4b. The edges of the blots are outlined with solid black lines, while the portions of the gels used for the figure are outlined with red lines.
